# A slow burn: Revisiting cross‐adaptation through long‐term heat acclimation

**DOI:** 10.1113/EP092883

**Published:** 2025-05-29

**Authors:** Elliott J. Jenkins, Michael Stembridge

**Affiliations:** ^1^ School of Sport and Health Sciences Cardiff Metropolitan University Cardiff UK

**Keywords:** cross adaptation, haemoglobin mass, heat stress, human/environmental and exercise physiology, hypoxia

1

Heat acclimtion has long been used to improve athlete tolerance and performance ahead of competition in warm–hot environments. Typically conducted across 5–14 days, repeated heat exposures improve thermoregulation while attenuating cardiovascular and perceptual strain. In recent years, research has shifted attention towards longer‐term heat acclimation interventions (≥5 weeks), which elicit additional physiological adaptations, most notably elevations in haemoglobin (Hb) mass that might contribute to improvements in maximal oxygen uptake (${\dot{V}}_{O_{2}\max}$) after 5 weeks of exercise in the heat (Lundby et al., 2023). The concept of cross‐adaptation, whereby adaptation to one environmental stressor (e.g., heat) induces beneficial physiological responses to another (e.g., hypoxia), has also garnered growing scientific interest. Previous research investigating cross‐adaptation has typically involved <10 heat exposures, demonstrating consistent reductions in physiological strain during submaximal exercise in hypoxia.

In this issue of *Experimental Physiology*, Rodrigues et al. (2025) advance this work by using a substantially longer, 6‐week postexercise hot‐water immersion (HWI) intervention to evaluate its impact on exercise performance in acute hypoxic conditions (O_2_ = 13%). The authors should be commended for extending the duration of intervention beyond previous studies, providing valuable insights into the temporal progression of cross‐adaptive responses. Primary findings demonstrate enhanced maximal exercise performance in acute hypoxia, characterized by increased aerobic peak power and absolute ${\dot{V}}_{O_{2}\max}$. Notably, participants also exhibited elevated Hb concentrations and reduced physiological strain during steady‐state exercise, evidenced by lower heart rates, reduced core temperatures, decreased ventilation and improved peripheral oxygen saturation ($S_{pO_{2}}$). Collectively, these adaptations illustrate the potential of prolonged heat acclimation to induce meaningful cross‐adaptation benefits to acute hypoxic stress.

Despite the increased Hb concentration observed by Rodrigues et al. (2025), the direct relevance of this adaptation for improving exercise performance in hypoxia merits further discussion. The theoretical model put forward by Wagner (1996) suggests that with increasing altitudes, Hb concentration and cardiac output contribute progressively less to ${\dot{V}}_{O_{2}\max}$, whereas pulmonary and muscular diffusion capacities become increasingly important. Supporting this empirically, Young et al. (1996) demonstrated erythrocyte infusion to be insufficient to ameliorate the decline in ${\dot{V}}_{O_{2}\max}$ at 4300 m a.s.l., suggesting that the performance‐enhancing effects of increased Hb concentrations are highly dependent upon the severity of arterial hypoxaemia. As such, although the elevated Hb levels reported by Rodrigues et al. (2025) might have contributed marginally to improved ${\dot{V}}_{O_{2}\max}$ at 13% O_2_, alternative mechanisms likely underpin their observed performance enhancements. Notably, given the relatively modest baseline fitness levels of participants (mean relative ${\dot{V}}_{O_{2}\max}$ = 47.7 mL kg^−1^ min^−1^), improvements in aerobic capacity attributable to a generalized training effect are probable, as reflected in the control group, who demonstrated an increase of 4.5 mL kg^−1^ min^−1^ over the 6‐week period. Additionally, physiological changes, such as reduced heart rates and core temperatures, might also explain observed performance benefits. The improvement in $S_{pO_{2}}$ after heat acclimation might have reduced the cardiac output requirement at a given submaximal workload. This enhancement in $S_{pO_{2}}$ is likely to be attributable, at least in part, to the observed reduction in core temperature after HWI. Decreased temperatures induce a leftward shift in the oxyhaemoglobin dissociation curve, increasing Hb–O_2_ affinity and enhancing oxygen loading in the lungs. Based on established temperature–affinity relationships, a 0.4°C reduction in core temperature [the same magnitude of change reported by Rodrigues et al. (2025)] would be expected to increase $S_{pO_{2}}$ by ∼1% at an inspired O_2_ fraction of 13%. Although this leftward shift might facilitate greater $S_{pO_{2}}$, it could concurrently impair oxygen unloading at the muscle, potentially limiting peripheral extraction. Consequently, we suggest that the practical applications of the prolonged heat acclimation protocol used by Rodrigues et al. (2025) might be most relevant at low‐to‐moderate altitudes (<2500 m a.s.l.), where the majority of sporting endeavours (apart from specialized mountaineering) typically occur.

Although Rodrigues et al. (2025) effectively controlled for the hydrostatic effects of water immersion by including a thermoneutral group, the independent influence of heat exposure is more difficult to isolate, given that both groups (consisting of relatively untrained individuals) underwent a new and structured exercise stimulus. Moving forwards, delineating the distinct physiological effects of heat stress, hydrostatic pressure and exercise would provide valuable insights. Acutely, HWI has been shown to stimulate erythropoiesis (DiMarco et al., 2024), yet the extent to which this response is driven by thermal stress, hydrostatic pressure or their interaction remains unclear. In contrast, the role of exercise appears more established, with both acute and prolonged bouts shown to increase erythropoietin independently of these stimuli, elevating circulating concentrations (Schwandt et al., 1991). Future research should therefore explore the magnitude of heat exposure required to stimulate erythropoiesis and Hb expansion optimally, compare different modes of passive heat exposure (e.g., HWI vs. sauna) systematically and clarify how concurrent exercise might modulate or amplify these responses. Targeted investigations in these areas would refine heat acclimation protocols and enhance their practical utility for improving athletic performance.

Although elevated Hb concentrations can contribute to enhanced arterial oxygen delivery, the lower heart rates observed during submaximal exercise by Rodrigues et al. (2025) suggest that cardiac adaptations might also have contributed to the observed improvements in ${\dot{V}}_{O_{2}\max}$. According to Fick's principle (that ${\dot{V}}_{O_{2}\max}$ is determined by maximal cardiac output and the difference in arteriovenous oxygen content), any increase in cardiac output would augment oxygen delivery directly and, in turn, ${\dot{V}}_{O_{2}\max}$. Although this study did not assess cardiac structure or function directly, previous work by Parsons et al. (2020) provides mechanistic insight into how short‐term heat acclimation can influence cardiovascular function. Following merely 5 days of exercise–heat exposure, significant increases in left ventricular end‐diastolic volume, stroke volume and left atrial volume were observed; changes indicative of enhanced preload and improved ventricular compliance, probably mediated by plasma volume expansion. Given that these adaptations occurred after only brief heat exposure, it is plausible that protocols exceeding 5 weeks (particularly in relatively untrained individuals) might induce more sustained cardiovascular remodelling, especially considering that previous long‐term heat acclimation studies have reported significant expansion in total blood volume (Lundby et al., 2023). Such changes would be expected to augment cardiac output further and, in turn, ${\dot{V}}_{O_{2}\max}$. However, the effects of long‐term heat acclimation on cardiac structure and function remain poorly characterized, and future research incorporating echocardiographic or cardiac MRI assessments would provide valuable insight into this potentially important, yet underexplored, area of adaptation.

In summary, Rodrigues et al. (2025) should be commended for their time‐intensive and innovative research, providing valuable insights into the physiological benefits of prolonged heat acclimation and its potential to induce meaningful cross‐adaptation effects in hypoxic conditions. Their novel use of postexercise HWI not only offers a practical, accessible, cost‐effective and environmentally friendly alternative to traditional heat‐acclimation protocols, but also highlights exciting avenues for future research and practical applications.

## AUTHOR CONTRIBUTIONS

Both authors have read and approved the final version of this manuscript and agree to be accountable for all aspects of the work in ensuring that questions related to the accuracy or integrity of any part of the work are appropriately investigated and resolved. All persons designated as authors qualify for authorship, and all those who qualify for authorship are listed.

## CONFLICT OF INTEREST

None declared.

## FUNDING INFORMATION

None.
